# Vertebrate bacterial gut diversity: size also matters

**DOI:** 10.1186/s12898-016-0071-2

**Published:** 2016-03-23

**Authors:** Jean-Jacques Godon, Pugazhendi Arulazhagan, Jean-Philippe Steyer, Jérôme Hamelin

**Affiliations:** UR0050, Laboratoire de Biotechnologie de l’Environnement, INRA, 102 avenue des étangs, 11100 Narbonne, France; Centre of Excellence in Environmental Studies, King Abdulaziz University, Jeddah, Saudi Arabia

**Keywords:** Biodiversity, Biogeography, Gut, Fingerprint, Species-area relationship

## Abstract

**Background:**

One of the central issues in microbial ecology is to understand the parameters that drive diversity. Among these parameters, size has often been considered to be the main driver in many different ecosystems. Surprisingly, the influence of size on gut microbial diversity has not yet been investigated, and so far in studies reported in the literature only the influences of age, diet, phylogeny and digestive tract structures have been considered. This study explicitly challenges the underexplored relationship connecting gut volume and bacterial diversity.

**Results:**

The bacterial diversity of 189 faeces produced by 71 vertebrate species covering a body mass range of 5.6 log. The animals comprised mammals, birds and reptiles. The diversity was evaluated based on the Simpson Diversity Index extracted from 16S rDNA gene fingerprinting patterns. Diversity presented an increase along with animal body mass following a power law with a slope *z* of 0.338 ± 0.027, whatever the age, phylogeny, diet or digestive tract structure.

**Conclusions:**

The results presented here suggest that gut volume cannot be neglected as a major driver of gut microbial diversity. The characteristics of the gut microbiota follow general principles of biogeography that arise in many ecological systems.

**Electronic supplementary material:**

The online version of this article (doi:10.1186/s12898-016-0071-2) contains supplementary material, which is available to authorized users.

## Background

Among a number of parameters, the ‘size’ of an ecosystem is often assumed to have a key impact on the management of diversity. In fact, the species-area relationship is central to the ecological theory [1] and was first described for macro-organisms [2]. For bacteria, the species-area relationship is generally expressed in terms of habitat volume (i.e., volume-area relationship) and has been illustrated in liquid sump tanks of metal-cutting machines [3], membrane bioreactors [4] and tree holes (i.e., rainwater accumulated in holes at the base of large trees) [5]. However, until present, the microbial species-volume relationship has never yet been studied for gut or body size, even though vertebrate gut size covers a wide range of magnitudes. There is a 10^6^ body mass factor between a tiny bird or a shrew and an elephant.

The vertebrate gut hosts a microbial community that fulfils many vital functions for the host: it enhances resistance to infection, stimulates mucosal immune defences, synthesizes essential vitamins and promotes caloric uptake by hydrolysing complex carbohydrates. The bacterial populations inhabiting the gut are complex, varying considerably from individual to individual and from species to species. However, gut microbial ecosystems are not a random association of microbes but are shaped by the host. A transfer occurs vertically from mothers to offspring or horizontally between individuals within a specific group. Such transfers have given rise to the long-standing co-evolution of microbiota and their hosts [6].

The benefit of bacterial diversity in the human gut has often been highlighted [7] and driving factors such as age [8], diverse lifestyles [9] and diet variations [10] have already been explored. Despite such an interest, the relationship between body mass and gut microbiota has never been explored whereas, in contrast, the positive links between the abundance of parasitic organisms or protozoal faunas and animal body size have been thoroughly referenced [11] [12]. The aim of the present study is to analyse a large bacterial dataset, comprising faeces collected from 71 different vertebrate species, in order to examine the effect of the volume-microbial diversity relationship in animal digestive tracts.

## Methods

### Sampling

All the animal samples were obtained from domesticated or captive populations in France (zoo, farm, aquarium, recreative farm or individual keeper). There is non-experimental research dedicated for this study, faeces samples were collected on ground with the animal keeper or animal owner without stresses for the animals. We obtained permissions from Lunaret zoo, Montpellier; Océanopolis, Brest; Réserve Africaine, Sigean; Mini Ferme Zoo, Cessenon sur Orb and consent from the animal owners (Jean-Philippe Steyer, Anais Bonnafous, Jean-Jacques Godon). Animal were living alone or in small groups (1 to 5). Furthermore, their food (meat, seeds, fruits or hay) were more standardized in comparison to wild diets.

Human stool specimens used in the present study were from infant and adult subjects included in international multicentric studies. Samples were collected between 2001 to 2005 and used on previous published studies. Infants samples were collected in the frame of the European project INFABIO (http://www.gla.ac.uk/departments/infabio/), ethical permission was obtained from Yorkhill Research Ethics Committee P16/03 and parents gave written informed consent [13]. Adults samples were collected in the frame of the European project Crownalife, the studies were approved by the Ethics Committee of Versailles Hospital Centre and written informed consent was obtained from all participants [14]. Approval for Institut National de la Recherche Agronomique to manage human-derived biological samples in accordance with Articles L.1243-3, R.1243-49 of “Code de la Santé Publique” was granted by the Ministry of Research and Education under number DC-2012-1728.

Faeces from 189 individuals belonging to 71 vertebrate species (31 mammals, 37 birds and 3 reptiles) were collected (Table 1). They were sub-divided into 80 categories according to species or to body mass (i.e., age (young–adult), sex (female–male), breed size (small–big–domesticated–wild), see Table 1). Body masses were provided by the breeder for large animals or obtained from literature for small animals. Body masses, along with diversity, were displayed with a logarithmic scale in order to highlight the linear shape of the power-law relationship. Except for the distinct dimorphism of male and female turkey samples, an average value of male and female body mass values was used. Dwarf or young individuals from the same species were also classified in specific body mass categories. For example, human samples were divided into two body mass categories: babies between 1 and 10 months old (mean of 5.8 kg) and adults between 29 and 61 years old (set at 70 kg). Composite faeces samples were avoided except for those that could not provide enough material for DNA extraction (less than 0.5 g).

**Table 1:** Animal data ranked by body mass

Name (common name)	Phylogeny	Body mass (kg)	Feeding type	Type of digestive tract	Size of animal husbandry group	Diversity	SD	Number of samples
*Taeniopygia guttata* (zebra finch)	Aves, Passeriformes	0.012	Granivorous	Hindgut colon	Large	1.2	0.1	3
*Serinus canaria* (canary)	Aves, Passeriformes	0.024	Granivorous	Hindgut colon	Large	1.6	0.5	2
*Ramphocelus bresilius* (brazilian tanager)	Aves, Passeriformes	0.035	Frugivorous	Hindgut colon	Small	3.4	0.4	4
*Melopsittacus undulatus* (budgerigar)	Aves, Psittaciformes	0.04	Granivorous	Hindgut colon	Large	1.9	3.0	3
*Ploceus cucullatus* (village weaver)	Aves, Passeriformes	0.04	Granivorous	Hindgut colon	Small	2.3	0.0	2
*Agapornis fischeri* (Fischer’s lovebird)	Aves, Psittaciformes	0.05	Granivorous	Hindgut colon	Large	1.5		1
*Agapornis roseicollis* (rosy-faced lovebird)	Aves, Psittaciformes	0.05	Granivorous	Hindgut colon	Large	2.1	0.0	2
*Amblyramphus holosericeus* (scarlet-headed blackbird)	Aves, Passeriformes	0.08	Carnivorous	Hindgut colon	Small	3.4	0.2	2
*Nymphicus hollandicus* (cockatiel)	Aves, Psittaciformes	0.08	Granivorous	Hindgut colon	Small	1.4	0.3	2
*Guira guira* (guira cuckoo)	Aves, Cuculiformes	0.14	Carnivorous	Hindgut colon	Small	2.9	0.5	4
*Poicephalus senegalus* (senegal parrot)	Aves, Psittaciformes	0.14	Granivorous	Hindgut colon	Small	2.6		1
*Streptopelia decaocto* (eurasian collard dove)	Aves, Columbidae	0.19	Granivorous	Hindgut colon	Large	2.4	0.5	3
*Corvus monedula* (eurasian jackdaw)	Aves, Passeriformes	0.22	Omnivorous	Hindgut colon	Small	1.8		1
*Psarocolius decumanus* (crested oropendola)	Aves, Passeriformes	0.3	Omnivorous	Hindgut colon	Small	3.8	0.3	3
*Columba livia* (pigeon)	Aves, Columbidae	0.3	Granivorous	Hindgut colon	Large	1.9		1
*Gallus gallus* (dwarf chicken)^a^	Aves, Galliformes	0.3	Granivorous	Hindgut caecum	Large	2.1	0.5	2
*Tauraco erythrolophus* (red-crested turaco)	Aves, Cuculiformes	0.35	Frugivorous	Hindgut colon	Small	3.8		1
*Agamia agami* (agami heron)	Aves, Ciconiiformes	0.46	Piscivorous	Hindgut colon	Small	3.6		1
*Coracopsis vasa* (vasa parrot)	Aves, Psittaciformes	0.5	Frugivorous	Hindgut colon	Small	2.4		1
*Chinchilla laniger xChinchilla brevicaudata* (chinchilla)	Mammalia, Rodentia	0.6	Herbivorous	Hindgut caecum	Small	4.2	0.1	2
*Ramphastos tucanus* (white-throated toucan)	Aves, Piciformes	0.675	Frugivorous	Hindgut colon	Small	3.6	0.4	3
*Chrysolophus pictus* (golden pheasant)	Aves, Galliformes	0.700	Granivorous	Hindgut caecum	Small	3.4		1
*Cavia porcellus* (domestic guinea pig)	Mammalia, Rodentia	0.8	Herbivorous	Hindgut caecum	Large	5.0	0.2	3
*Anas acuta* (northern pintail)	Aves, Anatidae	0.9	Granivorous	Hindgut caecum	Small	3.6		1
*Elaphe guttata* (corn snake)	Sauropsida, Serpentes	0.9	Carnivorous	Hindgut colon	Small	4.1		1
*Lampropeltis getula (*common kingsnake*)*	Sauropsida, Serpentes	1	Carnivorous	Hindgut colon	Small	3.3		1
*Ara ararauna* (blue-and-yellow macaw)	Aves, Psittaciformes	1	Granivorous	Hindgut colon	Small	2.8	0.1	2
*Anas platyrhynchos* (wild type duck)^a^	Aves, Anatidae	1.1	Granivorous	Hindgut caecum	Large	3.1		1
*Neochen jubata* (orinoco goose)	Aves, Anatidae	1.25	Granivorous	Hindgut caecum	Small	3.5		1
*Gallus gallus* (chicken)^a^	Aves, Galliformes	1.5	Granivorous	Hindgut caecum	Large	1.7	0.5	6
*Numida meleagris* (guinea-fowl)	Aves, Galliformes	2	Granivorous	Hindgut caecum	Large	3.0	0.7	3
*Branta sandvicensis* *(*nene)	Aves, Anatidae	2	Granivorous	Hindgut caecum	Small	2.6		1
*Oryctolagus cuniculus* (domestic rabbit)	Mammalia, Lagomorpha	2.2	Herbivorous	Hindgut caecum	Large	4.5	0.7	3
*Anas platyrhynchos* (domestic duck)^a^	Aves, Anatidae	2.3	Granivorous	Hindgut colon	Small	2.3	1.4	3
*Eudyptes chrysocome* (western rockhopper penguin)	Aves, Sphenisciformes	2.6	Piscivorous	Hindgut colon	Large	1.6	0.6	3
*Testudo hermanni boettgeri* (Hermann’s tortoise)	Sauropsida, Testudines	3	Herbivorous	Hindgut colon	Large	5.6		1
*Meleagris gallopavo* (turkey female)^a^	Aves, Galliformes	3	Granivorous	Hindgut caecum	Small	2.2		1
*Thylogale sp.* (pademelon)	Mammalia, Marsupials	3.5	Herbivorous	Hindgut colon	Small	4.3		1
*Cairina moschata* (muscovy duck)	Aves, Anatidae	4	Granivorous	Hindgut colon	Large	3.3		2
*Chauna torquata (*southern screamer*)*	Aves, Anseriformes	4	Herbivorous	Hindgut caecum	Small	3.4		1
*Canis lupus familiaris* (puppy)^a^	Mammalia, Carnivora	4	Carnivorous	Hindgut colon	Small	2.6		1
*Pavo cristatus* (blue peafowl)	Aves, Galliformes	5	Granivorous	Hindgut caecum	Small	3.7	0.1	2
*Anser anser domesticus* (domestic goose)	Aves, Anatidae	5	Granivorous	Hindgut caecum	Small	3.5	0.1	2
*Homo sapiens* (baby human caucasian)^a^	Mammalia, Primates	6	Omnivorous	Hindgut colon	Small	3.2	0.7	15
*Meleagris gallopavo* (turkey male)^a^	Aves, Galliformes	8	Granivorous	Hindgut caecum	Small	3.8		1
*Wallabia bicolor* (black wallaby)	Mammalia, Marsupials	9	Herbivorous	Hindgut colon	Small	4.8		1
*Hylobates lar* (gibbon)	Mammalia, Primates	10	Frugivorous	Hindgut colon	Small	5.5		1
*Aptenodytes patagonicus* (king penguin)	Aves, Sphenisciformes	13	Piscivorous	Hindgut colon	Small	2.8	1.1	4
*Capra hircus* (dwarf goat)	Mammalia, Ruminantia	15	Herbivorous	Ruminants foregut	Small	5.2	1.1	2
*Canis lupus familiaris* (medium size dog)^a^	Mammalia, Carnivora	20	Carnivorous	Hindgut colon	Small	3.2	1.1	2
*Ovis aries* (dwarf sheep)^a^	Mammalia, Ruminantia	20	Herbivorous	Ruminants foregut	Small	5.7	0.7	2
*Hippotragus equinus* (roan antelope)	Mammalia, Ruminantia	20	Herbivorous	Ruminants foregut	Small	5.7		1
*Tragelaphus streps* (greater kudu)	Mammalia, Ruminantia	20	Herbivorous	Ruminants foregut	Small	5.5		1
*Hystrix cristata* (crested porcupine)	Mammalia, Rodentia	25	Herbivorous	Hindgut caecum	Small	5.7		1
*Rhea americana* (greater rhea)	Aves, Rheiformes	31	Granivorous	Hindgut caecum	Large	4.0	0.5	4
*Ovis aries* (sheep)^a^	Mammalia, Ruminantia	40	Herbivorous	Ruminants foregut	Small	5.0		1
*Canis lupus familiaris* (big size dog)^a^	Mammalia, Carnivora	40	Carnivorous	Hindgut colon	Small	3.1		1
*Pan troglodytes* (chimpanzee)	Mammalia, Primates	40	Omnivorous	Hindgut colon	Small	5.3		1
*Dromaius novaehollandiae* (emu)	Aves, Casuariiformes	40	Granivorous	Hindgut colon	Small	3.9		1
*Capra hircus* (goat)	Mammalia, Ruminantia	50	Herbivorous	Ruminants foregut	Small	7.0		1
*Sus scrofa* (dwarf pig)^a^	Mammalia, Suina	55	Omnivorous	Hindgut colon	Small	5.4	0.4	2
*Lama glama* (llama)	Mammalia, Tylopoda	55	Herbivorous	Ruminants foregut	Small	5.4		1
*Homo sapiens* (adult human caucasian)^a^	Mammalia, Primates	70	Omnivorous	Hindgut colon	Large	4.4	0.8	34
*Sus scrofa* (pig)^a^	Mammalia, Suina	100	Omnivorous	Hindgut colon	Small	5.8	1.1	4
*Tragelaphus* *spekei* (sitatunga)	Mammalia, Ruminantia	100	Herbivorous	Ruminants foregut	Small	7.5		1
*Struthio camelus* (ostrich)	Aves, Struthioniformes	120	Herbivorous	Hindgut colon	Small	4.4	0.2	3
*Equus asinus* (donkey)	Mammalia, Equidae	150	Herbivorous	Hindgut caecum	Small	5.3	0.4	2
*Ammotragus lervia* (aoudad)	Mammalia, Ruminantia	150	Herbivorous	Ruminants foregut	Small	6.1		1
*Equus caballus* (pony)	Mammalia, Equidae	160	Herbivorous	Hindgut caecum	Small	5.6	0.1	2
*Panthera leo* (african lion)	Mammalia, Carnivora	160	Carnivorous	Hindgut colon	Small	4.4		1
*Equus zebra hartmannae* (mountain zebra)	Mammalia, Equidae	350	Herbivorous	Hindgut caecum	Small	5.4		1
*Syncerus caffer nanus* (forest buffalo)	Mammalia, Ruminantia	450	Herbivorous	Ruminants foregut	Small	2.9		1
*Camelus dromedarius* (arabian Camel)	Mammalia, Tylopoda	500	Herbivorous	Ruminants foregut	Small	3.2		1
*Bos grunniens* (yak)	Mammalia, Ruminantia	600	Herbivorous	Ruminants foregut	Small	5.3		1
*Tragelaphus oryx* (eland antelope)	Mammalia, Ruminantia	600	Herbivorous	Ruminants foregut	Small	6.2		1
*Bos taurus* (cow)	Mammalia, Ruminantia	750	Herbivorous	Ruminants foregut	Large	6.2	0.7	4
*Giraffa camelopardalis reticulata* (somali giraffe)	Mammalia, Ruminantia	1100	Herbivorous	Ruminants foregut	Small	6.4		1
*Giraffa camelopardalis peralta* (nigerian giraffe)	Mammalia, Ruminantia	1100	Herbivorous	Ruminants foregut	Small	6.6		1
*Ceratotherium simum* (white rhinoceros)	Mammalia, Rhinocerotidae	2500	Herbivorous	Hindgut colon	Small	5.6		1
*Elephas maximus* (asian elephant)	Mammalia, Proboscidea	3500	Herbivorous	Hindgut colon	Small	4.9		1

*SD* standard deviation

^a^ species with different sizes (young-adult, female-male, small-big or domesticated-wild)

### DNA extraction, PCR amplification and Capillary Electrophoresis Single Strand Conformation Polymorphism (CE-SSCP) fingerprinting

Genomic DNAs were extracted from 0.5 g of raw material using the procedure described by Godon et al. [15]. The V3 region of the 16S rRNA gene was amplified with *Bacteria*-specific primers and PCR products were analysed by CE-SSCP analysis using an ABI3130 Genetic Analyzer (Applied Biosystems, Foster City, CA, USA) in accordance with a previously described method [16]. All raw CE-SSCP data are available on Additional file 4.

### Calculation of diversity and statistical computing

Diversity was estimated by the Simpson Diversity Index from CE-SSCP fingerprinting patterns. The Simpson Diversity Index was expressed as $D = {1 \mathord{\left/ {\vphantom {1 {\sum {_{i = 1}^{p} a_{i}^{2} } }}} \right. \kern-0pt} {\sum {_{i = 1}^{p} a_{i}^{2} } }}$ where $a_{i}$ is the relative abundance of each CE-SSCP peak *p*. This index was directly calculated from each CE-SSCP fingerprint [17] using the R StatFingerprints library [18].

Preference was given to the Simpson Diversity Index from CE-SSCP fingerprinting rather than the Richness estimation because: (1) neither fingerprinting nor sequencing data can provide a robust estimation of richness [19]; (2) the Simpson Diversity Index can be estimated accurately with CE-SSCP fingerprinting [17, 20].

A generalized linear model was applied to fit the relationship between body mass and diversity. ANOVA followed by Tukey post hoc tests were used for determining the statistical difference between (sub-) categories and body mass or diversity, both expressed in a logarithmic scale. All statistics were performed under R software (version 3.1.2) [21]. The calculation of the slope *z* was based on the exponent of the power-law relationship as follows: diversity = *c* weight^*z.*^

## Results and discussion

**Fig. 1:**
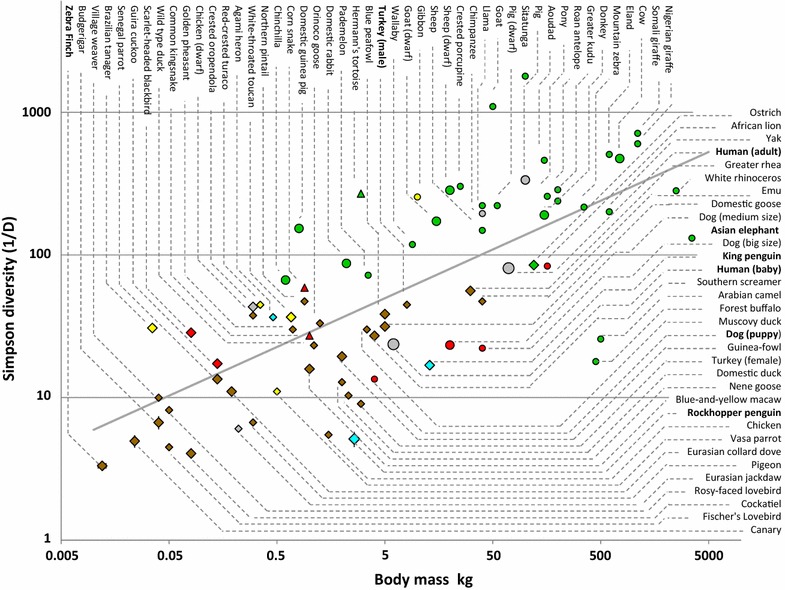
Relationship between the animal body mass and the Simpson Diversity Index for gut microbiota. *Diamonds*, *circles* and *triangles* correspond to birds, mammals and reptiles, respectively. *Small*, *medium* and *large* sizes correspond to 1, 2–5, >10 individuals, respectively. *Green*, *brown*, *grey*, *red*, *blue* and *yellow* colors correspond to herbivorous, granivorous, omnivorous, carnivorous, piscivorous and frugivorous diets, respectively. *Bold fonts* make reference to the animals mentioned in the text

The bacterial diversity of faeces from 189 vertebrates belonging to 71 species (31 mammals, 37 birds and 3 reptiles) was analysed (Table 1; Fig. 1). Analysis was only focused on diversity (Simpson Diversity Index), which can be accurately measured according to CE-SSCP fingerprinting patterns [15] (see the “Methods” section and Additional file 1). Apart from their phylogenetic position, animals can also be classified according to: (1) their diet (herbivorous, granivorous, omnivorous, carnivorous, piscivorous and frugivorous); (2) their metabolic body mass (from 12 g (zebra finch) to 3500 kg (Asian elephant)); (3) the structure of their digestive tracts; (4) and the size of the animal husbandry group (small and large). The present study focused on bacterial diversity, although changes within the structure of the bacterial communities were not taken into account. This study is also based on two assumptions: (1) the gut size should be proportional to the animal body mass, as has been demonstrated for herbivores [22] and birds [23]; and (2) the microbial diversity of faeces should be similar to that in the gut [24].

Results point to a correlation between animal body mass and microbial diversity (linear regression with a slope *z* of 0.338 ± 0.027; p value <2.2 × 10^−16^), irrespective of the diet, phylogeny or structure of the digestive tracts (Fig. 1). Consequently, the use of a greater amount of samples over a wider size range confirms previous works on unrelated bacterial communities that have suggested the existence of a link between volume and diversity in tree holes [5], membrane bioreactors [4] and metal-cutting fluid sump tanks [3]. In the present results, the Simpson Diversity Index ranges between 3.3 and 1789.5, thus corresponding to a 5.6 log body mass range (Fig. 1).

A wide variability in the diversity between individuals for a given species was observed. However the average diversity value for species that were represented by several individuals was close to the regression line (Fig. 1). For example, the average diversity value for adult human microbiota (34 samples) was 80.8 with a standard deviation of 294.2, and 23.7 ± 20.3 for the 15 baby human microbiota. As a matter of comparison, Trosvik et al. [25] observed a similar range of diversity (over 2 log-units of Shannon index) when analysing a time-series of 332 sequencings over 443 days, on a single male adult individual.

Animal gut microbiota covered a broad range of diversity ranging from 2.2 to 1808.0. This was comparable to the values found in various types of environment, like drinking water, raw milk, plant roots, activated sludge in wastewater treatment plants, compost or soil (Additional file 2). On one hand, the lowest diversity in gut microbiota varied around 2, similarly to those found in drinking water. On the other hand, the highest diversity in gut microbiota reaching about 1808 resembled the values found in soils (Additional file 2).

This vast range of variations in gut diversity is often associated with factors that are different to the body mass: diet [10], phylogeny [26], digestive tract structure [27], age [8] [28], way of life [29], ethnic origin [30], state of health (immune system, pregnancy, obesity) [31] [32], or genetic background [32]. Among these parameters, age has been well documented as the major one to explain these variations and the diversity or richness between human baby microbiota and those of adults [33] [34]. However the size of the gut also varies during infant growth. In this case, a difference in the microbial diversity between infant (29.9 ± 20.3) and adults (106.6 ± 76.0) was observed, concomitantly with changes in body mass when comparing human babies (6.5 ± 1.9) and adults (70 kg). The same observation was made for young and adult dog samples (Table 1). Furthermore, when comparing two penguin species that only differ in their body mass (only adult specimens, with the same diet and living in the same location), the relationship between microbial diversity and body mass still remain valid.

The correlation between body mass and diversity has been assessed for homogenous sub-categories (Table 2 and Additional file 3), thus excluding the potential effects of the different parameters. Indeed, the 189 samples could also be analysed according to phylogeny (reptile, bird, and mammal), diet (carnivorous, herbivorous, granivorous, omnivorous and piscivorous), gut structure (hindgut caecum, hindgut colon and foregut ruminant), age (baby and adult), and size of the animal husbandry group (small and large). Except for the latter category, all of them depended on the body mass (e.g. body mass was related to phylogeny, related to age or to ruminants). Significantly positive body mass/diversity correlations were observed for each sub-category, provided that a sufficient amount of data was available (over 50 samples minimum per sub-category) (Table 2; Additional file 3). The significant slopes z of the mass-diversity relationships generally ranged from 0.202 ± 0.043 to 0.380 ± 0.039. As the herbivorous group only contained 44 samples, the interestingly weak body mass diversity correlation with a *z* value of 0.137 could not be correctly interpreted.

**Table 2:** Bacterial diversity and animal weight within sub-categories, correlation between diversity and weight, and slope of the relationship of the diversity versus log-weight

Category		Number of samples	Simpson diversity mean (SD)	Weight in kg mean (SD)	Pearson correlation between diversity and weight		Power law relationship diversity = c weight^*z*^	
					cor	p value	Slope *z*	Confidence interval
Sub-categories								
Diet								
Carnivorous	13		32.0 (23.4)	19.0 (44.1)	0.277	0.359 (NS)	0.075 (NS)	–
Frugivorous	10		55.2 (68.5)	1.3 (3.1)	0.533	0.113 (NS)	0.234 (NS)	–
Granivorous	54		20.4 (21.7)	4.4 (9.4)	0.667	3.7e−08 (***)	0.298 (***)	0.205–0.391
Herbivorous	44		301.6 (342.3)	354.5 (668.1)	0.338	0.025 (*)	0.137 (*)	0.018–0.256
Omnivorous	60		111.3 (149.0)	50.5 (32.4)	0.542	7.7e−06 (***)	0.361 (***)	0.214– 0.508
Piscivorous	8		20.4 (20.8)	7.5 (5.9)	0.030	0.944 (NS)	0.029 (NS)	–
Phylogeny								
Bird	85		25.1 (23.6)	7.8 (23.0)	0.456	1.1e−05 (***)	0.202 (***)	0.116–0.288
Mammal	101		194.9 (268.8)	183.3 (464.0)	0.415	1.6e−05 (***)	0.272 (***)	0.153–0.391
Reptile	3		119.3 (131.9)	1.6 (1.2)	0.964	0.172 (NS)	1.686 (NS)	–
Gut structure								
Caecum	46		70.4 (80.6)	26.1 (65.3)	0.528	1.7e−04 (***)	0.397 (***)	0.203–0.591
Colon	124		78.5 (122.5)	79.0 (382.7)	0.678	<2.2e−16 (***)	0.293 (***)	0.236–0.350
Rumen	19		484.9 (449.7)	411.8 (384.5)	−0.036	0.883 (NS)	−0.031 (NS)	–
Group size								
Large	85		93.1 (151.0)	65.8 (156.4)	0.734	1.3e−15 (***)	0.380 (***)	0.303–0.457
Small	104		137.2 (253.7)	130.6 (449.1)	0.632	6.6e−13 (***)	0.300 (***)	0.227–0.372
Age								
Adult	173		125.6 (222.2)	110.3 (364.6)	0.687	<2.2e−16 (***)	0.337 (***)	0.283–0.391
Baby	16		28.9 (20.1)	6.3 (2.0)	0.205	0.446 (NS)	0.427 (NS)	–
All	189		117.4 (214.3)	101.5 (350.0)	0.675	<2.2e−16 (***)	0.338 (***)	0.284–0.391

*NS* not significant

* low significance, *** high significance

The observed slope *z* was similar to that reported for ‘island’ patterns of bacterial diversity such as metal-cutting fluid sump tanks (*z* = 0.245–0.295) [3] and tree holes (*z* = 0.26) [5] and varied within a similar range to that reported for plants and animals from discrete islands (*z* = 0.25–0.35). The slope *z*-values reported for continuous patterns (such as marsh sediment [35] with *z*-values between 0.02 and 0.04) are generally much lower than those reported for discrete habitats.

According to these results, which confirm the assumption that species and volume are related, guts can compared to an archipelago, where microbes originating from feed tend to colonise the available niches provided by the gut. This is also in line with the MacArthur and Wilson biogeography theory [1]. Size, similarly to island environments appears to reflect the heterogeneity of the environment. Hence, a large gut size should provide more space, enabling a large microbial diversity to settle in [36].

## Conclusions

The aim of this study was not to explain the genesis of bacterial diversity in vertebrate guts but was rather focused on producing evidence on the role of gut size in the maintenance of a level of microbial diversity. This work highlights the hitherto unexplored relationship between volume and diversity in the case of gut microbiota. Gut volume should henceforth be taken into account along with other parameters to explain the level of diversity. Finally, this work confirms the relevance of the microbial world when addressing ecological issues such as the relationship between species diversity and the size of the habitat [37].

## Availability of supporting data

Our data are provided in the electronic supplementary materials (Additional file 4).

## Additional files

10.1186/s12898-016-0071-2 Examples of representative CE-SSCP fingerprinting patterns from low-diverse to high-diverse samples.
10.1186/s12898-016-0071-2 Simpson Diversity Index calculated from 671 CE-SSCP fingerprinting patterns. Samples were grouped according to their ecosystem of origin.
10.1186/s12898-016-0071-2 Simpson Diversity Index as a function of animal weight for (A) all the samples and for the sub-categories; (B) diet; (C) phylogeny; (D) gut structure; (E) age and (F) group size. The slope of the linear regression is indicated.
10.1186/s12898-016-0071-2 Raw 671 CE-SSCP profiles. Columns correspond to the samples. The 7359 lines correspond to the fluorescence intensity according to time.

## Abbreviations

CE-SSCP: capillary electrophoresis single strand conformation polymorphism
PCR: polymerase chain reaction
16S rRNA: 16S ribosomal RNA
ANOVA: analysis of variance
