# Supplementary material for: Vertebrate bacterial gut diversity: size also matters
Source: BMC Ecol. 2016 Mar 23;16:12. doi: 10.1186/s12898-016-0071-2 (PMC4804487; doi:10.1186/s12898-016-0071-2)

Additional file 1:
Examples of representative CE-SSCP fingerprinting patterns from low-diverse to high-diverse samples.

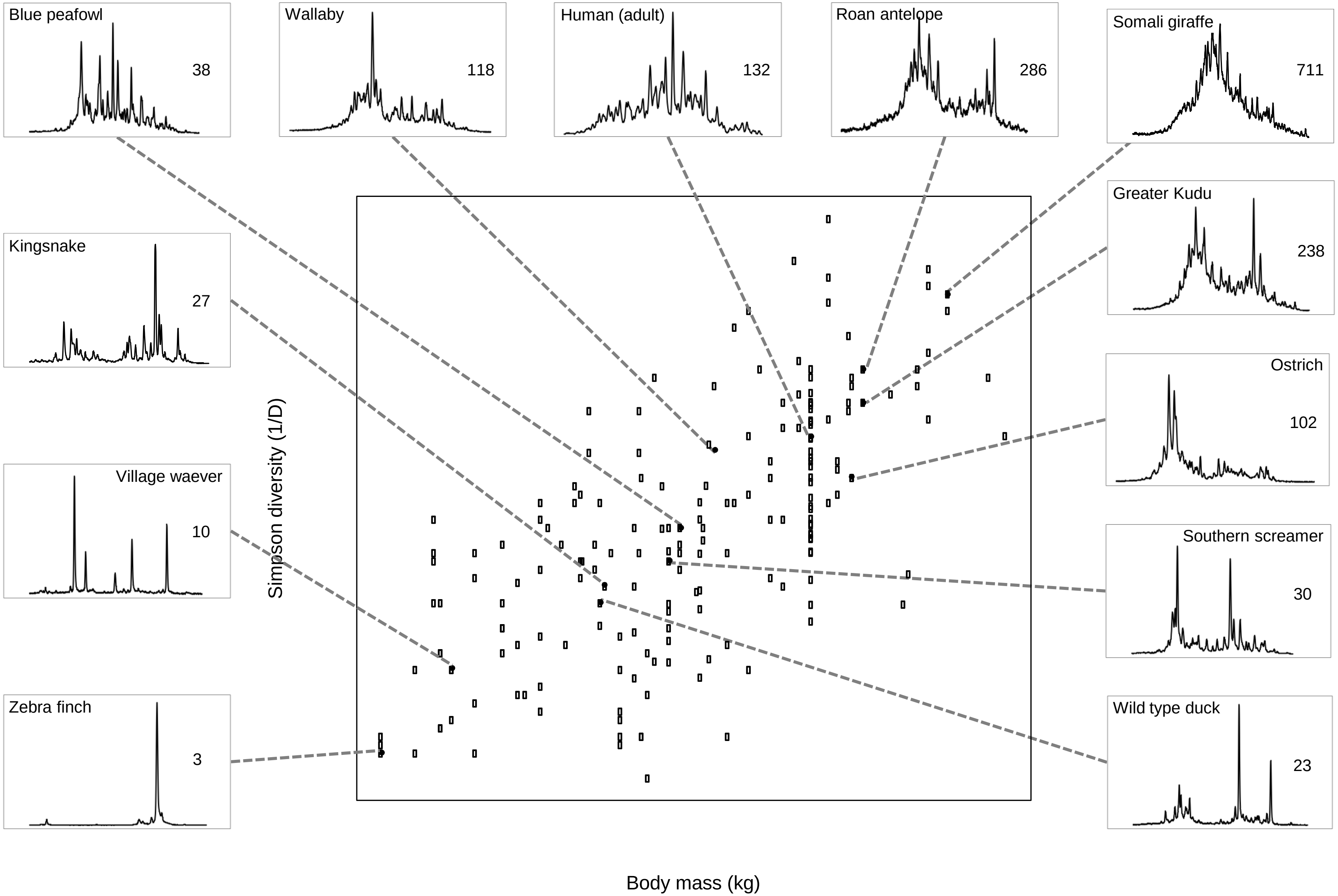

Supplement: Supplementary file 1 — 10.1186/s12898-016-0071-2 Examples of representative CE-SSCP fingerprinting patterns from low-diverse to high-diverse samples. [file 12898_2016_71_MOESM1_ESM.pdf]
