# Supplementary material for: Vertebrate bacterial gut diversity: size also matters
Source: BMC Ecol. 2016 Mar 23;16:12. doi: 10.1186/s12898-016-0071-2 (PMC4804487; doi:10.1186/s12898-016-0071-2)

Additional file 2:
Simpson Diversity Index calculated from 671 CE-SSCP fingerprinting patterns. Samples were grouped according to their ecosystem of origin (Y-axis) and the number of samples from each ecosystem is indicated in bracket after the ecosystem name. Boxes represent dispersion of data from the first quartile to the third quartile, with median inside. Standard deviation above and below the mean of the data is displayed as doted lines. Boxplot is ordered with increasing median value of diversity.

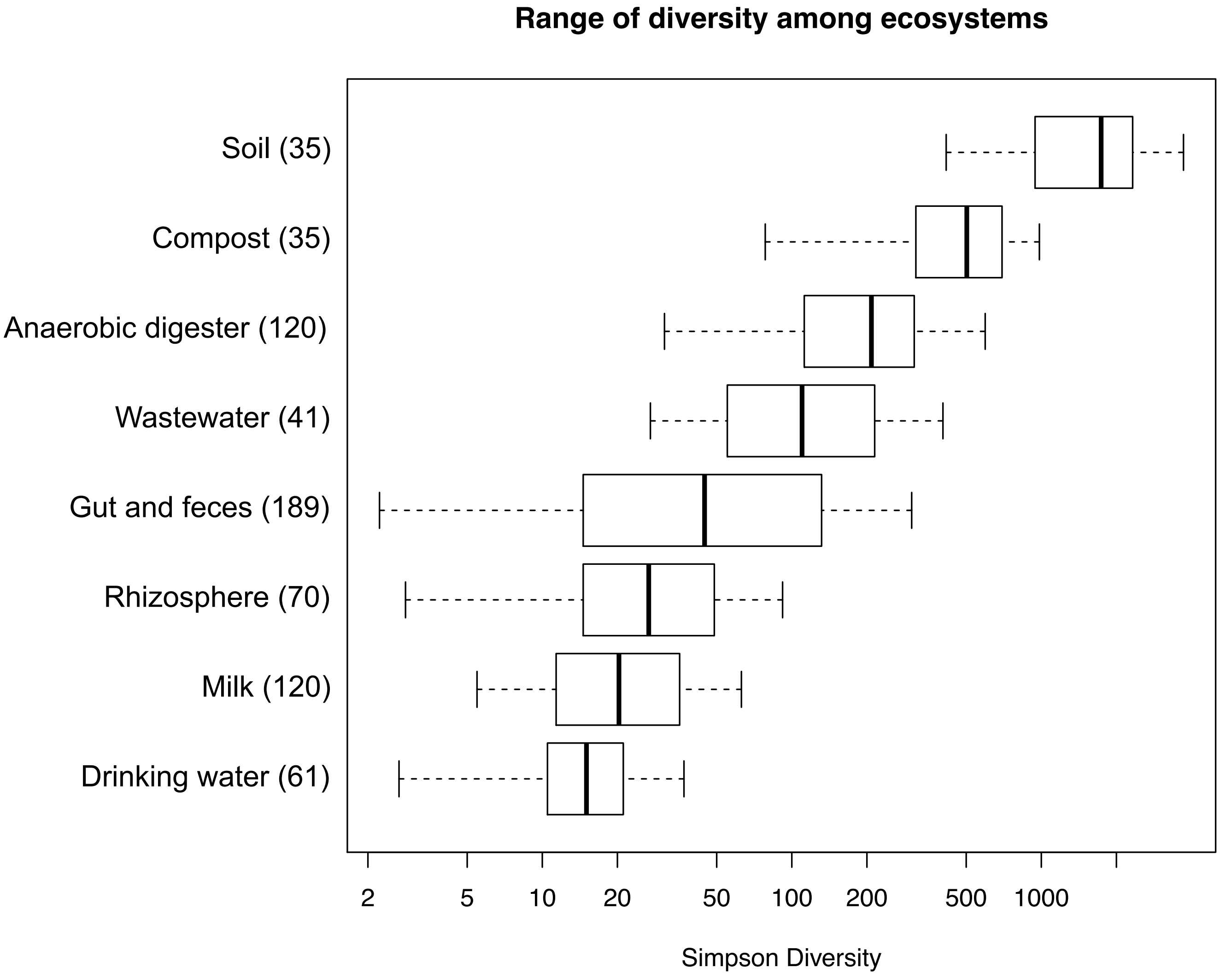

Supplement: Supplementary file 2 — 10.1186/s12898-016-0071-2 Simpson Diversity Index calculated from 671 CE-SSCP fingerprinting patterns. Samples were grouped according to their ecosystem of origin. [file 12898_2016_71_MOESM2_ESM.pdf]
