# Supplementary material for: Vertebrate bacterial gut diversity: size also matters
Source: BMC Ecol. 2016 Mar 23;16:12. doi: 10.1186/s12898-016-0071-2 (PMC4804487; doi:10.1186/s12898-016-0071-2)
Supplement: Supplementary file 3 — 10.1186/s12898-016-0071-2 Simpson Diversity Index as a function of animal weight for (A) all the samples and for the sub-categories; (B) diet; (C) phylogeny; (D) gut structure; (E) age and (F) group size. The slope of the linear regression is indicated. [file 12898_2016_71_MOESM3_ESM.pdf]

Additional file 3:
Simpson Diversity Index as a function of animal weight for (A) all the samples and for the sub-categories; (B) diet; (C) phylogeny; (D) gut structure; (E) age and (F) group size. The power law relationship $\log_{diversity} = c \log_{weight}^{z}$ is displayed as a red ligne. Intercept *c* and slope *z* of the regression are indicated at the bottom of each panel, with the range of values (p-value 0.05) in brackets. The prevision and confidence intervals of the linear regression are displayed as dashed and dotted lines respectively.

(A) All the samples

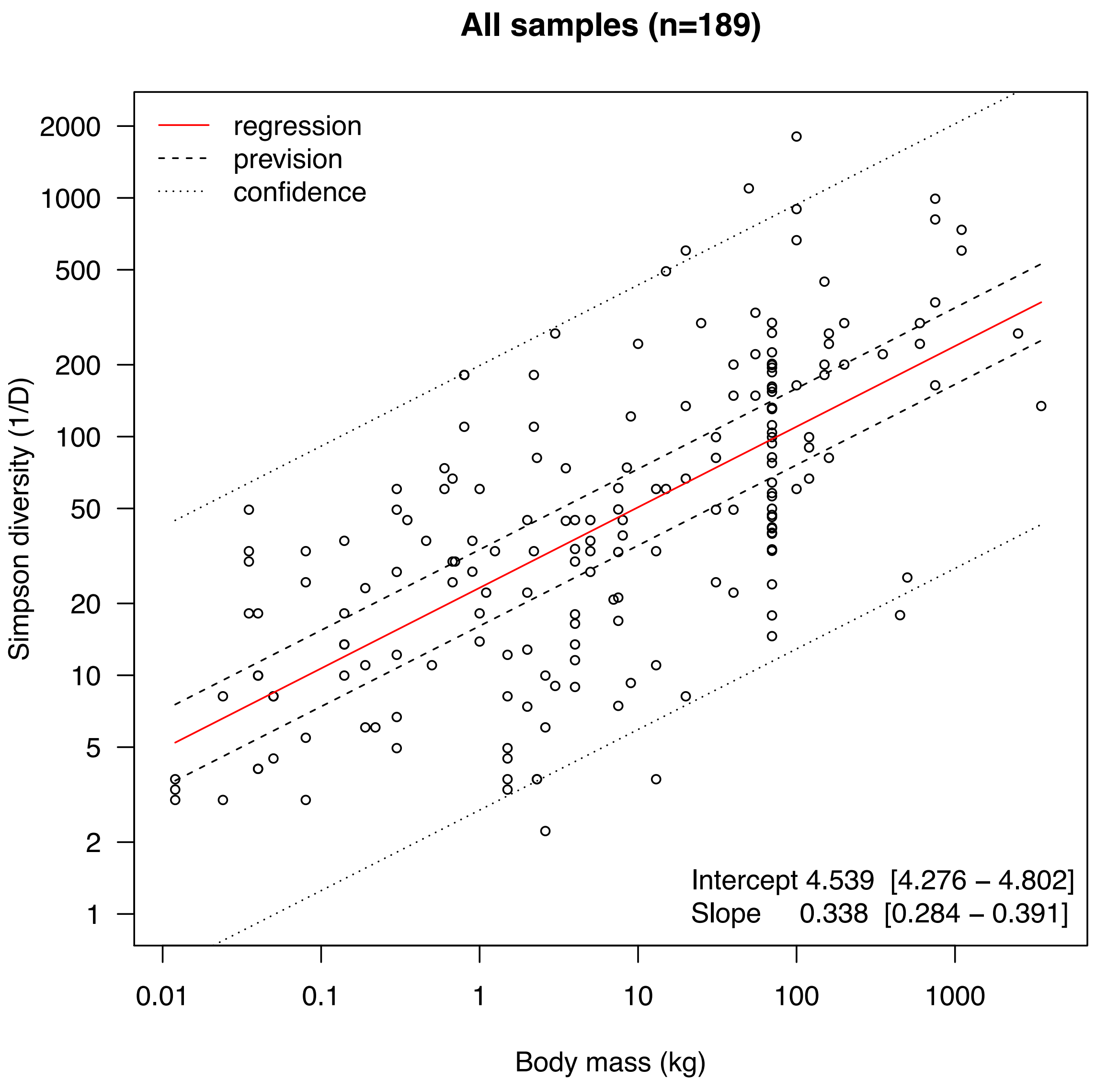

(B) Diet

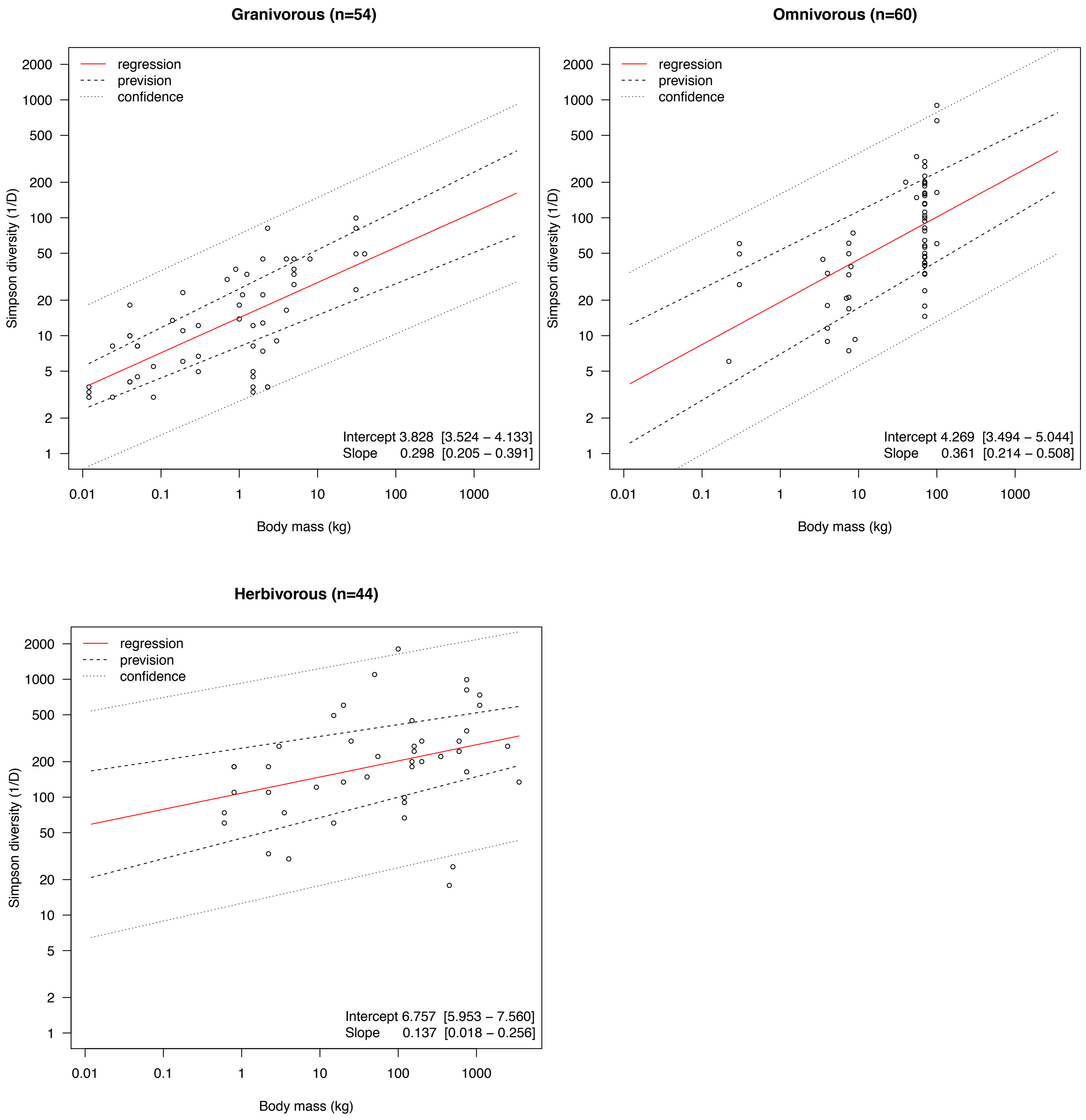

Granivorous (n=54)
regression
prevision
confidence
Intercept 3.828 [3.524 – 4.133]
Slope 0.298 [0.205 – 0.391]
Simpson diversity (1/D)
Body mass (kg)
Omnivorous (n=60)
regression
prevision
confidence
Intercept 4.269 [3.494 – 5.044]
Slope 0.361 [0.214 – 0.508]
Simpson diversity (1/D)
Body mass (kg)
Herbivorous (n=44)
regression
prevision
confidence
Intercept 6.757 [5.953 – 7.560]
Slope 0.137 [0.018 – 0.256]
Simpson diversity (1/D)
Body mass (kg)

(C) Phylogeny

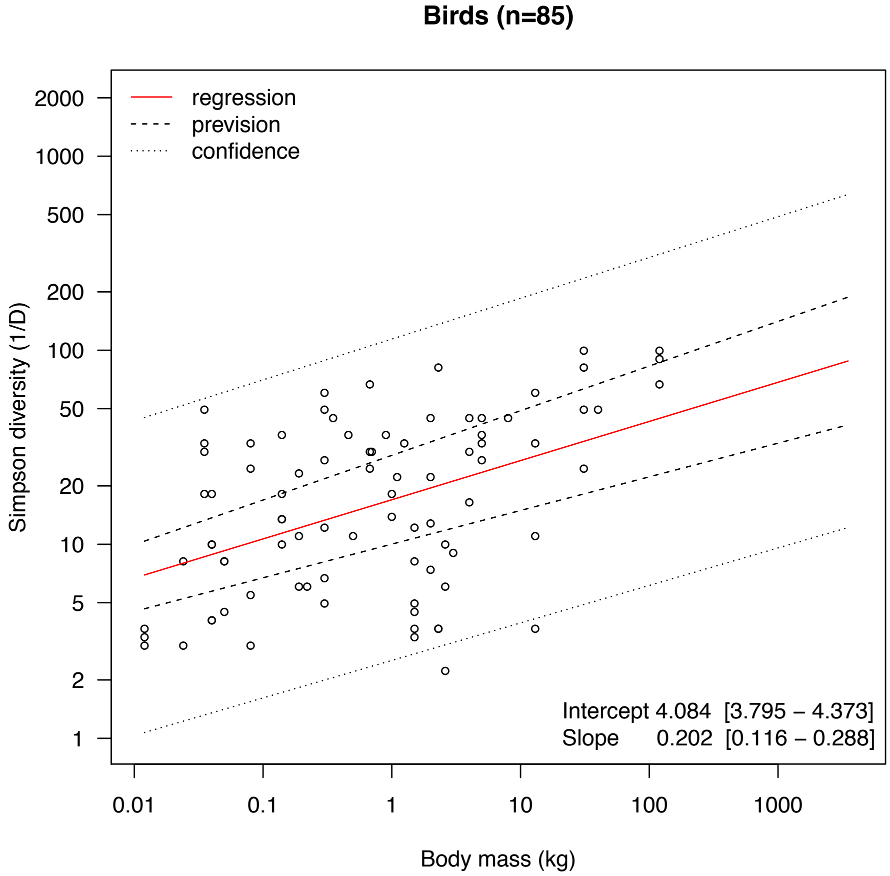

Birds (n=85)
regression
prevision
confidence
Intercept 4.084 [3.795 – 4.373]
Slope 0.202 [0.116 – 0.288]
Simpson diversity (1/D)
2000
1000
500
200
100
50
20
10
5
2
1
0.01
0.1
1
10
100
1000
Body mass (kg)

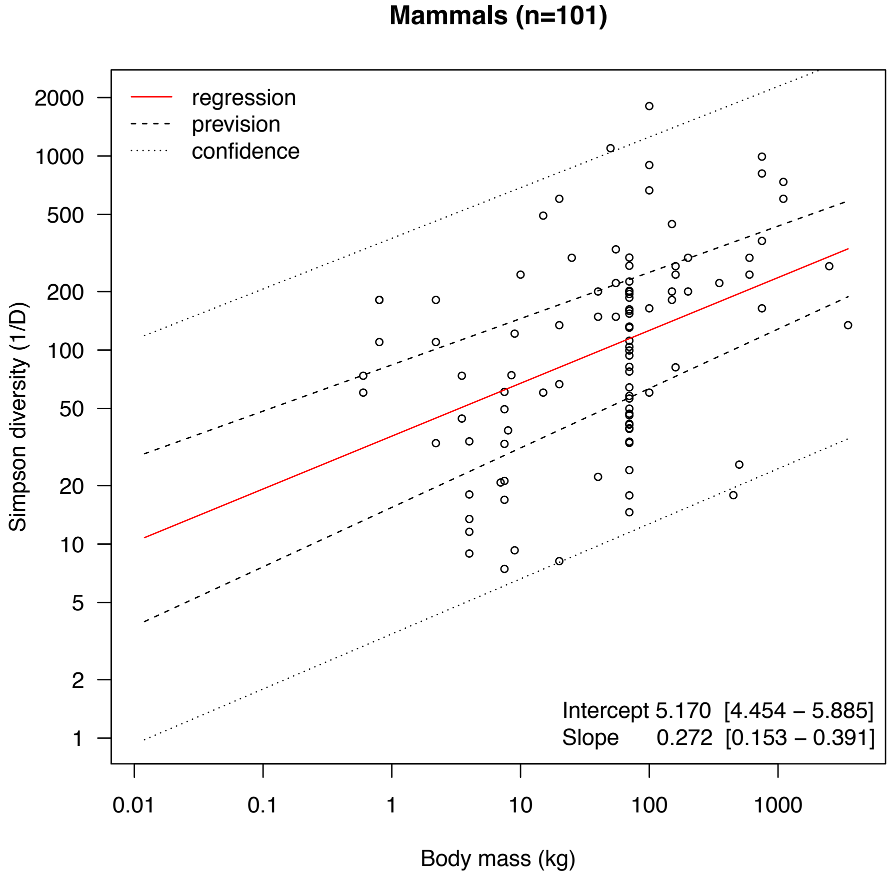

Mammals (n=101)
regression
prevision
confidence
Intercept 5.170 [4.454 – 5.885]
Slope 0.272 [0.153 – 0.391]
Simpson diversity (1/D)
2000
1000
500
200
100
50
20
10
5
2
1
0.01
0.1
1
10
100
1000
Body mass (kg)

(D) Gut structure

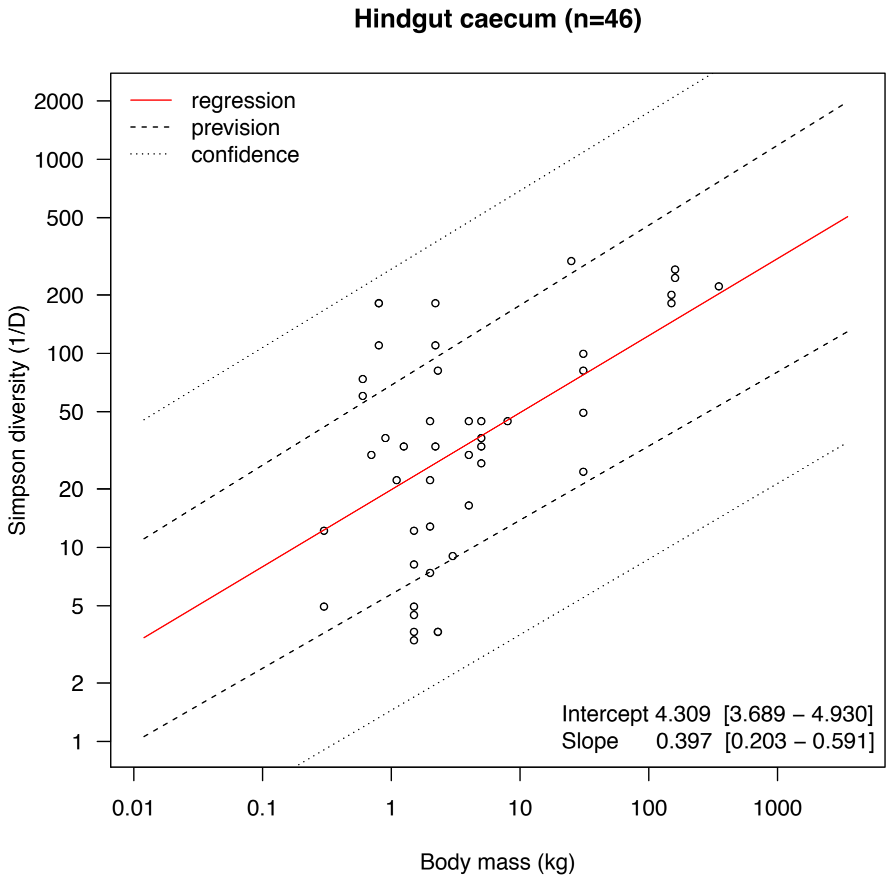

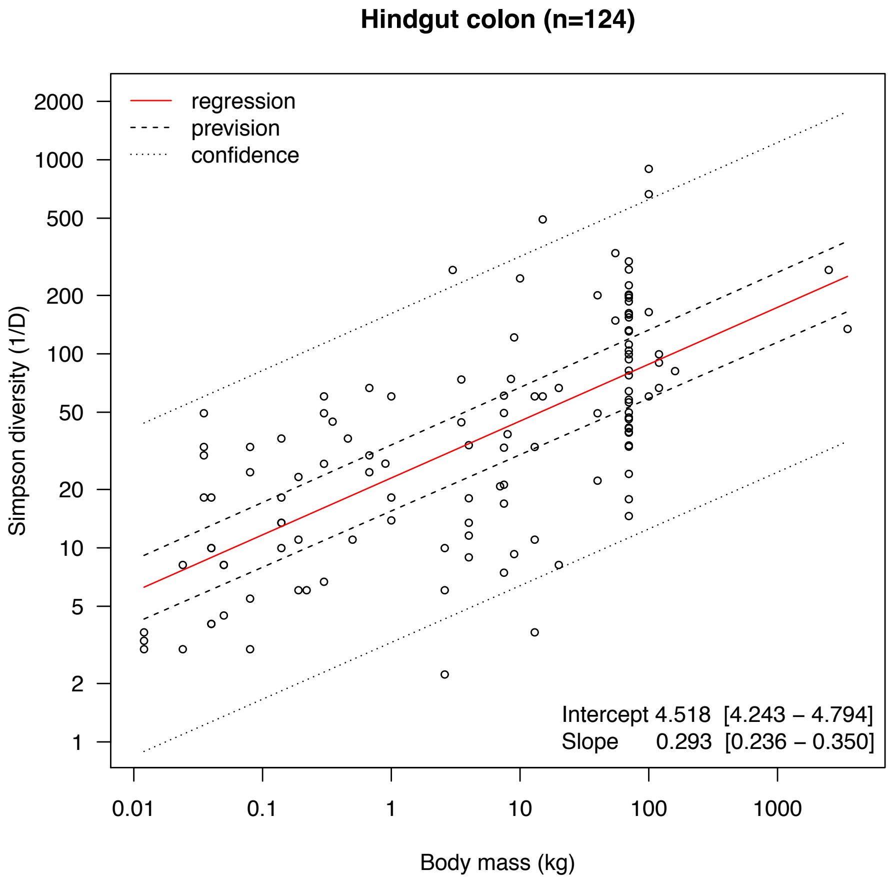

(E) Age

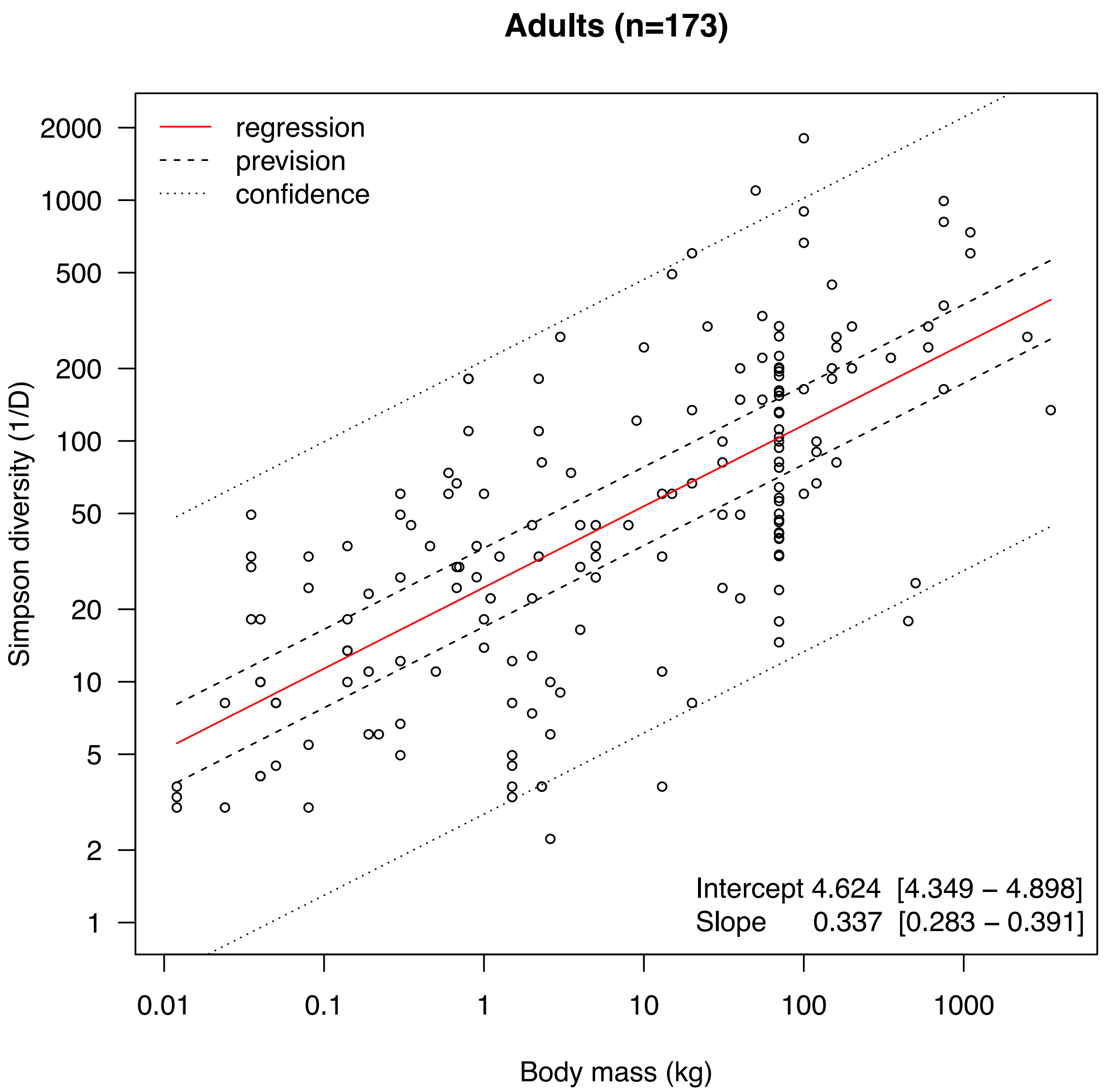
Adults (n=173)
regression
prevision
confidence
Intercept 4.624 [4.349 – 4.898]
Slope 0.337 [0.283 – 0.391]
Simpson diversity (1/D)
2000
1000
500
200
100
50
20
10
5
2
1
0.01
0.1
1
10
100
1000
Body mass (kg)

(F) Group size

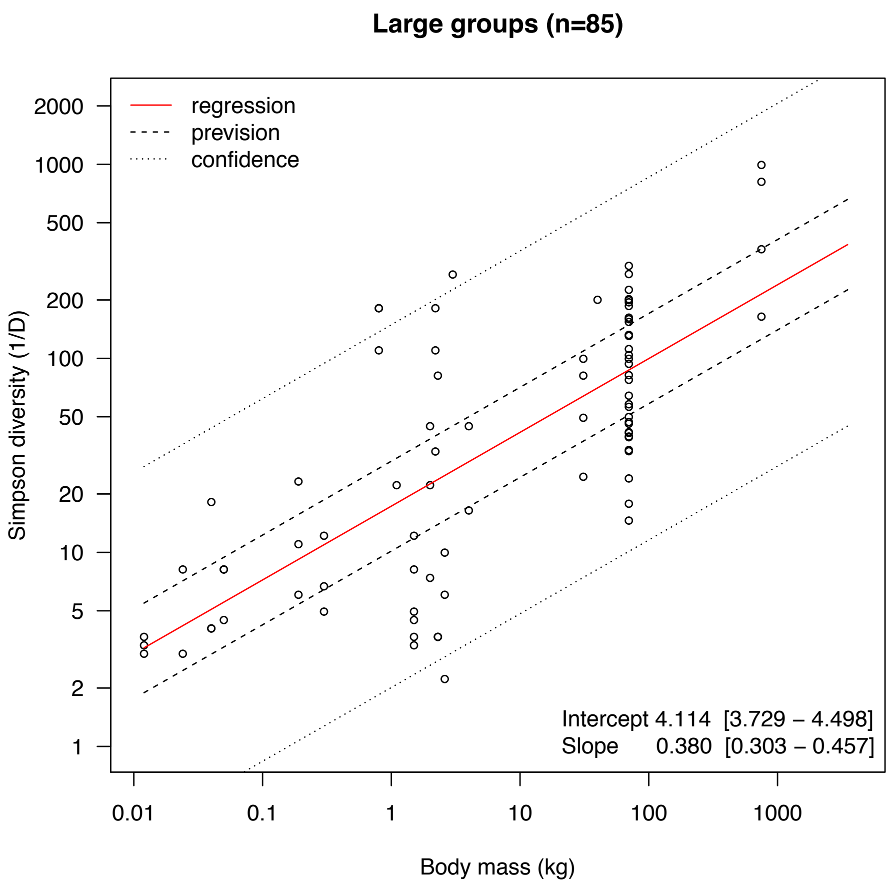
Large groups (n=85)
regression
prevision
confidence
Intercept 4.114 [3.729 – 4.498]
Slope 0.380 [0.303 – 0.457]
Simpson diversity (1/D)
2000
1000
500
200
100
50
20
10
5
2
1
0.01
0.1
1
10
100
1000
Body mass (kg)

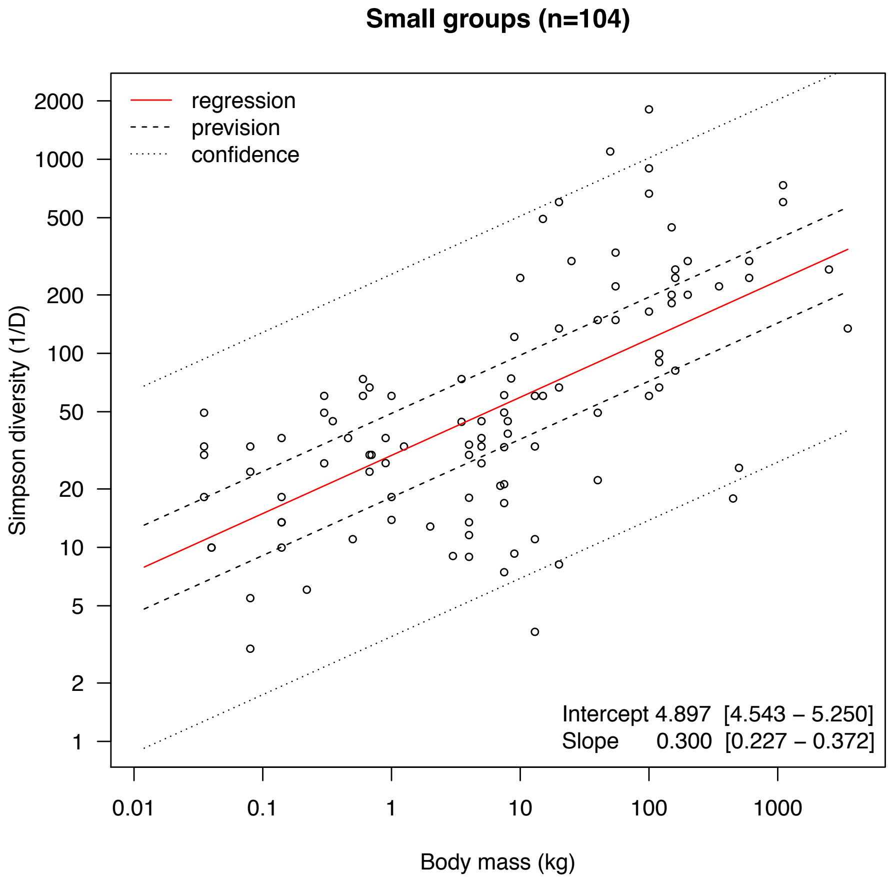
Small groups (n=104)
regression
prevision
confidence
Intercept 4.897 [4.543 – 5.250]
Slope 0.300 [0.227 – 0.372]
Simpson diversity (1/D)
2000
1000
500
200
100
50
20
10
5
2
1
0.01
0.1
1
10
100
1000
Body mass (kg)
